# Phase angle as a potential marker of early nutritional and metabolic changes following metabolic bariatric surgery: a retrospective study

**DOI:** 10.3389/fnut.2026.1853597

**Published:** 2026-09-09

**Authors:** Elif Mizrak, Halime Pulat Demir

**Affiliations:** 1 Department of Nutrition and Dietetics, College of Health Sciences, Istanbul Medipol University, Istanbul, Türkiye; 2 Department of Nutrition and Dietetics, Faculty of Health Sciences, Istanbul Beykent University, Istanbul, Türkiye

**Keywords:** bariatric surgery, metabolic surgery, nutritional biomarker, obesity, phase angle

## Abstract

**Background:**

The phase angle (PhA) is associated with nutritional status and serves as a marker of cellular and overall health. The aim of this study is to evaluate changes in PhA during the early postoperative period (6 months) in patients undergoing metabolic bariatric surgery (MBS), and to identify potential factors correlated with these changes.

**Methods:**

This retrospective study included 120 patients who underwent MBS (Sleeve Gastrectomy, *n* = 6; Transit Bipartition, *n* = 114). Biochemical parameters and anthropometric measurements were collected at four time points: preoperatively and at the 1st, 3rd, and 6th postoperative month follow-up visits.

**Results:**

PhA values at 1-, 3-, and 6-months postoperatively were significantly lower than preoperative values (*p* < 0.05). PhA was inversely correlated with body mass index (BMI), fat mass, and fat percentage, and positively correlated with muscle mass, fat-free mass (FFM), and total body water (TBW) (*p* < 0.001). Among these, fat mass showed the strongest negative correlation, while TBW exhibited the strongest positive correlation across all time periods. A significant negative correlation was observed between the amount of change in PhA at 6 months and age (*r* = −0.283, *p* = 0.002), while height was positively correlated with the change in PhA at 6 months (*r* = 0.261, *p* = 0.004). Ferritin (*r* = −0.358, *p* < 0.001) and vitamin D levels (*r* = −0.234, *p* = 0.010) were both negatively correlated with the amount of change in PhA at 6 months. In multivariate linear regression analysis adjusting for age, sex, and BMI, vitamin D, and excess weight loss (EWL) remained independently associated with the 6-month change in PhA, while Homeostatic Model Assessment for Insulin Resistance (HOMA-IR) showed a borderline significant association (*p* = 0.046).

**Conclusion:**

This study suggests that PhA significantly decreases during the early postoperative period following MBS and is associated with several body composition and metabolic parameters, with vitamin D and EWL emerging as independent predictors of this decline and a borderline association observed for HOMA-IR. These findings suggest that PhA may serve as a potential adjunctive marker reflecting early nutritional and metabolic adaptations in this patient population.

## 1 Introduction

Metabolic bariatric surgery (MBS) is widely recognized as an effective treatment for severe obesity; however, the evaluation of postoperative nutritional and metabolic status remains challenging (1, 2). Traditional measures such as body mass index (BMI) are insufficient to reflect changes in body composition and cellular health (3, 4). Bioelectrical Impedance Analysis (BIA) has emerged as a practical alternative for assessing body composition beyond BMI (5, 6). Therefore, alternative biomarkers that can sensitively capture these changes are needed (7, 8).

The phase angle (PhA) derived from BIA is an indicator of membrane damage and body cell mass, associated with clinical deterioration and mortality in various diseases (9, 10). Since it is obtained by measuring the resistance and reactance of tissues to current flow, PhA is considered a reliable indicator of hydration status and body cell mass (11). In clinical practice, lower PhA values have been consistently associated with compromised nutritional status (11, 12). In healthy individuals, PhA ranges between 6° and 7° and can reach 8.5° in athletes. A low PhA (<5°) indicates a loss of cellular integrity (11). Vassilev et al. reported 3.9° for PhA as most suitable cut-off for a successful operation in bariatric patients (13).

The determinants of PhA in healthy individuals are age, sex, and BMI (5). Height may influence PhA measurements, as bioelectrical impedance measurements are inherently dependent on conductor length. Therefore, normalization for height provides a qualitative measure of soft tissue that does not depend on body size (14). PhA decreases with increasing age as body water decreases in parallel with the increase in body fat mass and decrease in muscle mass (5). As a result of the increase in muscle and fat masses, PhA increases as BMI increases, but this correlation was observed in individuals with BMI < 30 kg/m^2^, while an inverse correlation was found in severely obese individuals with BMI > 40 kg/m^2^ (7, 14). Thus, PhA, as an indicator of body cell mass and cell integrity, may change in obesity and obesity-related diseases, a condition characterized by changes in muscle structure and function (7, 15).

Several studies have reported a reduction in PhA following MBS (11–14); however, Vassilev et al. (13) observed a decrease in PhA that was not statistically significant. Studies comparing PhA across different surgical techniques have indicated that patients who undergo Roux-en-Y Gastric Bypass (RYGB) may be at higher risk of malnutrition and tend to have lower PhA compared to those who undergo Sleeve Gastrectomy (SG) (16–18).

While previous studies in the literature have primarily demonstrated a decrease in PhA following MBS and have focused on comparisons with a limited set of parameters, most of this research has been conducted in patients undergoing RYGB and/or SG (13, 16, 18). The present study included patients who underwent transit bipartition (TB) (*n* = 114), a surgical technique that has been less frequently investigated in the context of PhA, alongside a smaller group of SG patients (n = 6). Furthermore, studies evaluating PhA in relation to a broad range of body composition and metabolic parameters across multiple postoperative time points remain scarce. The present study aims to address this gap by evaluating changes in PhA during the early postoperative period in patients undergoing MBS, and by identifying potential factors associated with these changes.

## 2 Materials and methods

### 2.1 Study design

This was a retrospective study conducted in patients aged 18–65 years with at least grade II obesity (BMI ≥ 35 kg/m^2^) who were admitted for MBS (Sleeve Gastrectomy and Transit Bipartition) between January 2021 and April 2023, and who regularly attended postoperative follow-up visits at first, third and sixth months. Initial approval for this study was obtained from the Ethics Committee of the Institute of Graduate Programs at Bilgi University, as documented in the “Ethics Committee Evaluation Result” dated April 24, 2023 (No. 2023–20,160-081). Subsequently, institutional permission was granted by the hospital through a consent form, permitting the use of data from patients who underwent surgery with these two techniques, provided that all personal information remained confidential, and this study was conducted in accordance with the Declaration of Helsinki.

### 2.2 Study population and data collection

**Figure 1:**
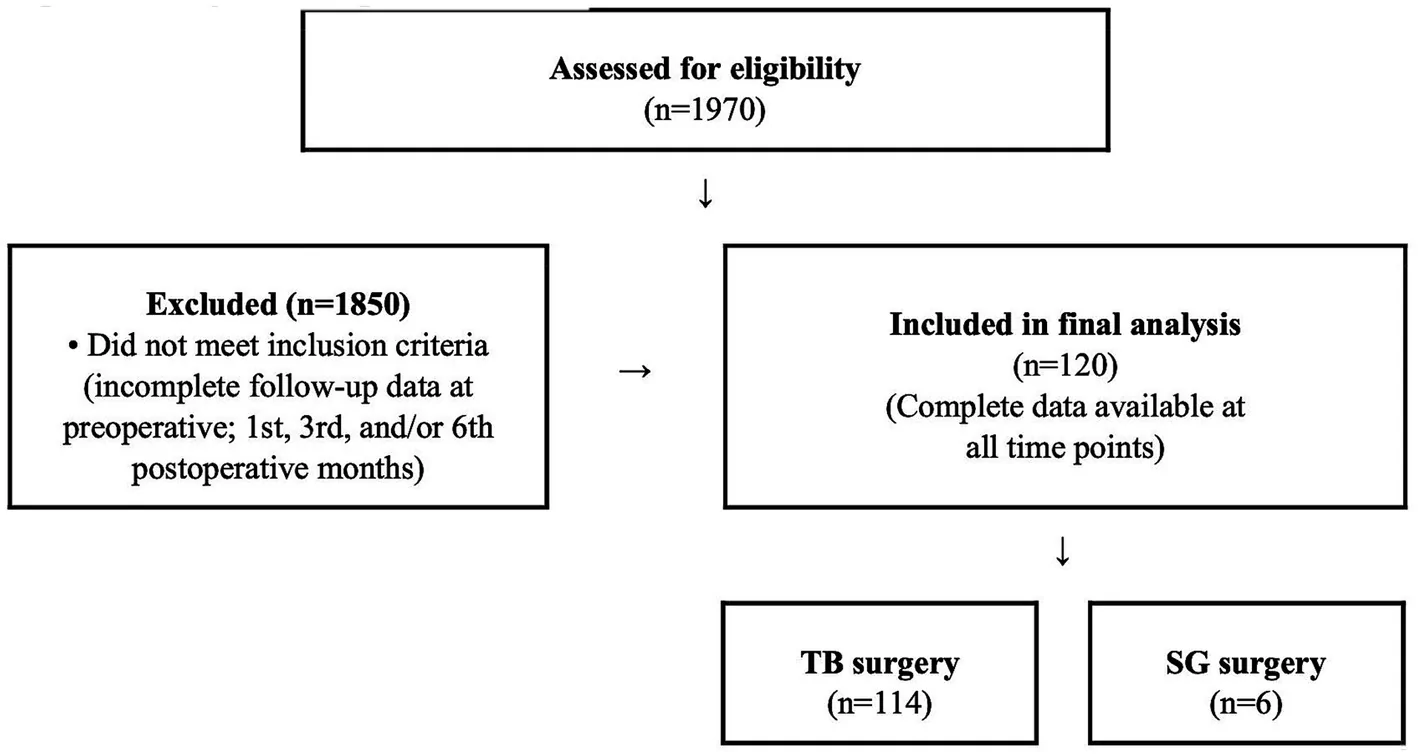
Study flow diagram. TB, Transit bipartition; SG, Sleeve gastrectomy.

The required sample size was calculated as 122 participants using the G*Power program, based on a medium effect size (0.3) and a study power of 0.95. Ultimately, a total of 1,970 patients underwent SG or TB surgery within the specified period; however only 120 patients who attended regular follow-ups were enrolled in the study. The study flow diagram was prepared in accordance with the STROBE reporting guidelines (Figure 1).

Patients with preoperative micronutrient deficiencies received additional supplementation prior to surgery as planned by the physician. In the early postoperative period, all patients were prescribed a standardized multivitamin supplementation protocol: liquid form for the first month and capsule form through the end of the third month.

The postoperative dietary progression followed a standardized institutional protocol consistent with guidelines (19): from a liquid diet to a pureed diet, and advancing to soft/solid foods by weeks five and six after surgery. During this initial six-week period, patients were advised to target protein intake of at least 60 g/day, and adequate hydration (1.5 L/day) in line with standard recommendations after MBS (20). After the six-week period, patients were guided by the team’s dietitian, who provided individualized nutritional counseling and/or plans as necessary, tailored to each patient’s tolerance, weight loss progress, and dietary habits. Dietary assessments were conducted for postoperative follow-up.

Biochemical parameters and anthropometric measurements were collected at four time points: preoperatively and at the 1st, 3rd, and 6th postoperative month follow-up visits. To improve clinical interpretability, changes between baseline and postoperative 6th month were specifically analyzed, as this time point represents a critical phase of rapid metabolic adaptation. The change in PhA (ΔPhA) was calculated as the 6-month value minus the preoperative value (ΔPhA = PhA₆ₘₒₙₜₕ − PhA_baseline); thus, a positive value indicates an increase and a negative value indicates a decrease in PhA over the follow-up period. Anthropometric measurements included weight (kg), height (m), body mass index (BMI, kg/m^2^), fat mass (kg), fat percentage (%), muscle mass (kg), fat-free mass (FFM, kg), and total body water (TBW, %). Phase angle (PhA, °) and all anthropometric measurements except height were assessed using the SECA MBCA 515 Medical Body Composition Analyzer. Measurements were performed after 8–12 h of fasting, with patients barefoot and wearing light clothing without metal objects. Height was measured at baseline using a stadiometer with patients barefoot.

Biochemical parameters, including HbA1c, fasting plasma glucose (FPG), homeostasis model assessment of insulin resistance (HOMA-IR), total cholesterol, high-density lipoprotein (HDL), low-density lipoprotein (LDL), triglycerides, alanine transaminase (ALT), aspartate aminotransferase (AST), gamma-glutamyl transferase (GGT), creatinine, and urea, as well as nutrition-related parameters such as vitamin B_12_, vitamin D, ferritin, parathormone (PTH), hemoglobin (HGB), and hematocrit, were monitored by collecting blood samples at each period.

### 2.3 Study outcomes

The primary outcome was to determine the variation in phase angle among MBS patients during the early postoperative period. The secondary outcome was to identify which factors potentially associated with changes in phase angle in patients who have undergone MBS.

### 2.4 Statistical analysis

Preoperative and postoperative 1st, 3rd, and 6th month data of the patients’ anthropometric measurements and biochemical parameters were evaluated. Descriptive statistics include the mean, standard deviation, median, minimum, maximum, frequency, and proportion values. The normality of the distribution of variables was assessed using the Kolmogorov–Smirnov, as the primary criterion, with the Shapiro–Wilk tests as supplementary check. The Mann–Whitney U test was used to compare non-normally distributed quantitative independent data. The Wilcoxon signed-rank test was used to analyze paired quantitative data. The Friedman test was used to evaluate longitudinal changes across repeated measurements. The Chi-square test was used to analyze qualitative independent data. Pearson’s correlation coefficient was used to assess relationships between PhA and variables meeting the normality assumption, while Spearman’s rank correlation coefficient was applied for variables that did not meet this assumption. Given the large number of correlations examined, no correction for multiple comparisons was applied; therefore, correlation findings should be considered exploratory, and marginal *p*-values should be interpreted with caution. A multivariate linear regression analysis was performed using the Enter method to identify variables independently associated with the 6-month change in PhA (ΔPhA) as the dependent variable. Age, sex, and BMI were included as covariates, while muscle mass, FPG, HOMA-IR, ferritin, vitamin D, and EWL were included as candidate predictors based on their significant univariate correlation with 6-month change in PhA (Table 1); height and body weight, although significantly correlated with 6-month change in PhA, were not entered separately given their inherent relationship with BMI. Multicollinearity was assessed using variance inflation factor (VIF) values, with VIF < 5 indicating the absence of significant multicollinearity. As candidate predictors for this model were themselves selected on the basis of univariate correlations without correction for multiple comparisons, the regression findings should likewise be regarded as exploratory rather than confirmatory. A *p*-value of less than 0.05 was considered statistically significant. All analyses were performed using SPSS version 27.0.

**Table 1:** The amount of change in PhA according to all parameters.

	Amount of change in PhA at 6th month	
	r	*p*
Age (years)	−0.283	**0.002**
Height (cm)	0.261	**0.004**
Amount of change at 6th month		
Body weight (kg)	0.180	**0.050**
BMI (kg/m^2^)	0.291	**0.001**
Fat mass (kg)	−0.034	0.714
Fat percentage (%)	0.146	0.112
Muscle mass (kg)	0.212	**0.020**
FFM (kg)	0.128	0.165
TBW (%)	0.088	0.339
HbA1c	0.136	0.140
FPG	0.255	**0.005**
HOMA-IR	0.181	**0.048**
Creatinine	−0.150	0.102
Urea	0.025	0.784
ALT	−0.154	0.092
AST	−0.136	0.138
GGT	−0.124	0.178
HGB	0.168	0.067
HCT	0.098	0.289
Total cholesterol	0.024	0.792
LDL	−0.047	0.609
HDL	0.022	0.813
Triglycerides	−0.035	0.707
Ferritin	−0.358	**<0.001**
Parathormone	−0.047	0.614
Vitamin B_12_	−0.005	0.953
Vitamin D	−0.234	**0.010**
EWL	−0.220	**0.016**

Spearman Correlation, ALT: Alanine Aminotransferase, AST: Aspartate Aminotransferase, EWL: excess weight loss, FPG: fasting plasma glucose, GGT: Gamma-Glutamyl Transferase, HCT: Hematocrit, HDL: High-Density Lipoprotein, HGB: Hemoglobin, HOMA-IR: homeostatic model assessment of insulin resistance, LDL: Low-Density Lipoprotein, TBW: total body water. Bold *p*-values indicate statistical significance (*p* < 0.05).

## 3 Results

### 3.1 Characteristics of the study population

The study consisted of 120 patients, 56 male (46.7%) and 64 female (53.3%), with a mean age of 46.7 ± 9.8 years. No statistically significant differences were found between the TB and SG groups in terms of age, height, sex distribution, or diabetes mellitus status (*p* > 0.05; Table 2). The longitudinal changes in biochemical and anthropometric parameters across all follow-up time points (preoperative, 1st, 3rd, and 6th months) are presented in Supplementary Table 1.

**Table 2:** Comparison of characteristics of population study between the TB and SG groups.

		TB (n:114)				SG (n:6)				*p*	
		Mean±SD/n-%			Median	Mean±SD/n-%			Median		
Age (years)		46.7 ± 9.8			48.0	47.7 ± 13.6			48.0	0.861	m
Sex	Female	61		53.5%		3		50.0%		0.867	X^2^
	Male	53		46.5%		3		50.0%			
Height (cm)		165.5 ± 10.6			164.0	167.8 ± 14.3			166.0	0.833	m
Preoperative anthropometric measurements											
Weight (kg)		110.8 ± 17.9			110.1	133.2 ± 35.2			139.8	0.095	m
BMI (kg/m^2^)		40.4 ± 4.7			38.9	46.6 ± 6.8			46.7	***0.016***	m
Fat percentage (%)		44.8 ± 6.1			45.5	49.8 ± 5.1			50.4	0.062	m
Fat mass (kg)		49.4 ± 10.5			46.5	65.9 ± 17.2			64.2	***0.010***	m
Muscle mass (kg)		29.7 ± 7.1			28.8	32.3 ± 13.1			31.4	0.866	m
FFM (kg)		61.2 ± 12.4			60.1	67.3 ± 20.6			68.9	0.523	m
TBW (%)		40.9 ± 4.6			40.4	35.2 ± 6.2			36.6	***0.037***	m
Preoperative biochemical parameters											
HbA1c (%)		7.6 ± 2.0			7.1	8.4 ± 3.7			7.2	0.928	m
FPG (mg/dL)		158.7 ± 71.8			138.6	169.8 ± 101.3			141.7	0.866	m
HOMA-IR		9.0 ± 9.0			6.6	6.1 ± 3.1			7.1	0.434	m
Creatinine (mg/dL)		0.79 ± 0.21			0.78	0.98 ± 0.43			0.84	0.312	m
Urea (mg/dL)		29.6 ± 8.9			28.0	39.4 ± 22.5			32.4	0.309	m
ALT (U/L)		40.8 ± 34.0			31.0	26.8 ± 13.5			23.0	0.159	m
AST (U/L)		29.0 ± 18.7			24.6	23.5 ± 15.0			17.4	0.116	m
GGT (U/L)		52.0 ± 58.5			37.0	57.5 ± 62.5			30.6	0.876	m
Total cholesterol (mg/dL)		210.5 ± 54.7			206.2	206.7 ± 33.2			196.4	0.942	m
LDL (mg/dL)		135.3 ± 43.1			129.2	131.0 ± 31.8			129.2	0.928	m
HDL (mg/dL)		48.1 ± 12.2			45.1	50.7 ± 9.8			52.3	0.516	m
Triglycerides (mg/dL)		213.8 ± 132.8			176.5	152.8 ± 109.0			98.4	0.119	m
HGB (g/dL)		14.0 ± 1.8			14.0	13.3 ± 2.1			13.9	0.500	m
HCT (%)		40.9 ± 6.0			41.6	39.4 ± 4.6			40.7	0.396	m
Ferritin (ng/mL)		89.1 ± 96.7			48.7	66.6 ± 45.9			68.5	0.923	m
PTH (pg/mL)		61.7 ± 30.6			54.9	98.7 ± 62.6			76.6	0.082	m
Vitamin B12		477.2 ± 320.6			385.5	427.0 ± 144.7			372.5	0.966	m
Vitamin D		14.7 ± 8.1			13.1	7.9 ± 3.2			8.0	***0.009***	m
DM	(−)	78		68.4%		3		50.0%		0.389	X^2^
	(+)	36		31.6%		3		50.0%			

^m^Mann–Whitney U test, ^X2^ Chi-square test; ALT, Alanine Aminotransferase; AST, Aspartate Aminotransferase; DM, Diabetes Mellitus; FPG, Fasting plasma glucose; GGT, Gamma-Glutamyl Transferase; HCT, Hematocrit; HDL, High-Density Lipoprotein; HGB, Hemoglobin; HOMA-IR, homeostatic model assessment of insulin resistance; LDL, Low-Density Lipoprotein; TBW, total body water; PTH, parathormone; SD, Standard deviation. Bold *p*-values indicate statistical significance (*p* < 0.05).

### 3.2 Change of phase angle

Phase angle (PhA) values were consistently higher in the TB group than in the SG group across all time points; however, these differences did not reach statistical significance (all *p* > 0.05). Over the follow-up period, a gradual decline in PhA values was observed in both groups, with no statistically significant between-group differences detected at any time point (*p* > 0.05; Table 3).

**Table 3:** Variation in PhA.

	TB (n:114)		SG (n:6)		*p*	
	Mean. ± SD	Median	Mean±SD	Median		
PhA						
Preoperative (t0)	5.5 ± 0.6	5.4	4.8 ± 1.0	4.9	0.080	m
Post-op 1. Month (t1)	5.1 ± 0.6	5.0	4.7 ± 0.8	4.8	0.332	m
Post-op 3. Month (t2)	4.9 ± 0.6	4.9	4.6 ± 0.8	4.7	0.469	m
Post-op 6. Month (t3)	4.8 ± 0.7	4.7	4.5 ± 0.9	4.7	0.531	m

^m^ Mann–Whitney U test. Bold *p*-values indicate statistical significance (*p* < 0.05).

**Figure 2:**
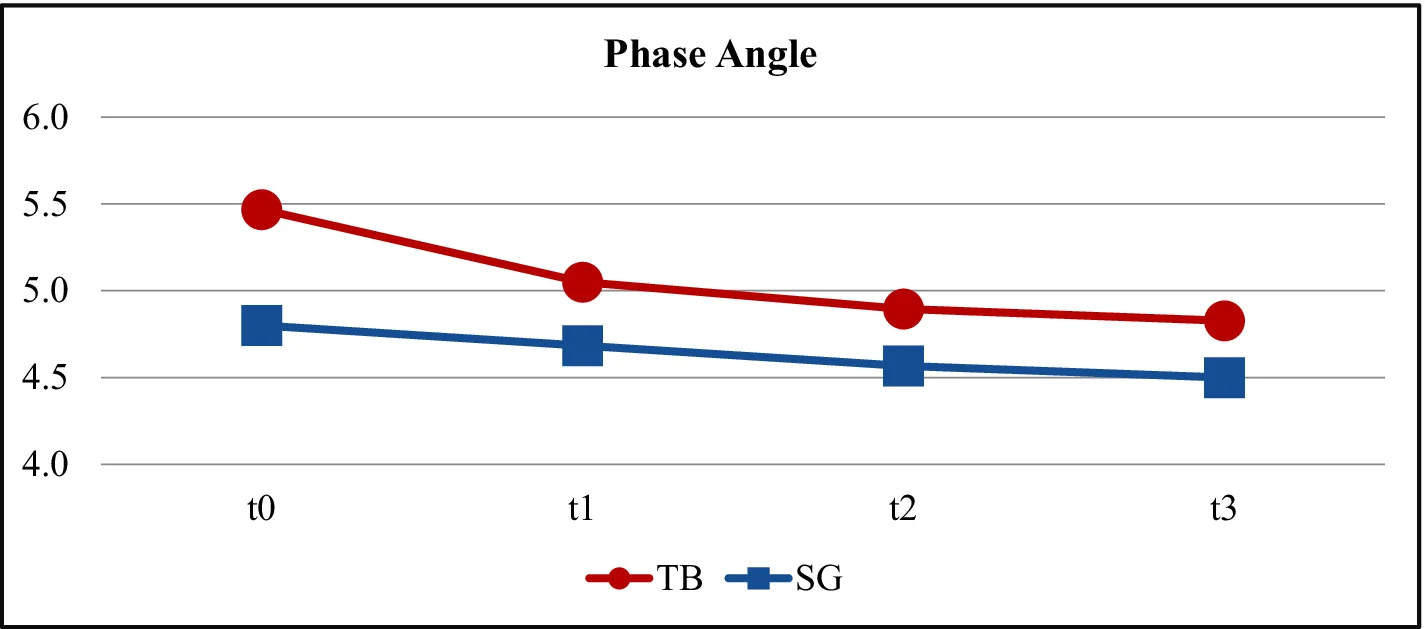
The variation in PhA over time. t0 = preoperative, t1 = postoperative 1st month, t2 = postoperative 3rd month, t3 = postoperative 6th month, TB = transit bipartition, SG = sleeve gastrectomy. The SG trajectory (*n* = 6) is presented for descriptive purposes only, given the small sample size, and is not intended for inferential comparison with the TB group.

PhA values at 1-, 3-, and 6-months postoperatively were significantly lower than preoperative values (*p* < 0.05) (Figure 2). Additionally, PhA continued to decrease over time: values at 3 and 6 months were significantly lower than at 1 month, and values at 6 months were significantly lower than at 3 months (*p* < 0.05 for all comparisons; Table 4). Overall longitudinal analysis using the Friedman test showed a significant decrease in PhA throughout follow-up [*χ*^2^ (3) = 182.875, *p* < 0.001].

**Table 4:** The amount of change of PhA.

	Min-Max.	Median	Mean±SD	p*	p**	p***
PhA						
t0	3.00 − 6.90	5.40	5.44 ± 0.64			
t1	3.20 − 6.50	5.00	5.03 ± 0.60	***<0.001***		
t2	3.00 − 6.20	4.85	4.88 ± 0.64	***<0.001***	***<0.001***	
t3	2.90 − 6.30	4.70	4.81 ± 0.66	***<0.001***	***<0.001***	***0.044***

^w^Wilcoxon test/p* Change in phase angle based on preoperative findings, p** Change in phase angle based on postoperative 1st month findings, p*** Change in phase angle based on postoperative 3th month findings. Bold *p*-values indicate statistical significance (*p* < 0.05).

### 3.3 Phase angle and its correlations with biochemical and anthropometric measurements

Phase angle (PhA) demonstrated consistent and statistically significant correlations with body composition parameters at all time points. Specifically, PhA was inversely correlated with BMI, fat mass, and fat percentage, and positively correlated with muscle mass, FFM, and TBW (*p* < 0.001). Among these, fat mass showed the strongest negative correlation, while TBW exhibited the strongest positive correlation (Table 5).

**Table 5:** The relationship between PhA and anthropometric measurements in all periods.

	PhA			
	t0	t1	t2	t3
Weight (kg) (r)	0.083	0.059	−0.026	0.108
BMI (kg/m^2^) (r)	−0.314**	−0.291**	−0.267**	−0.088
Fat mass (kg) (r)	−0.642**	−0.529**	−0.462**	−0.355**
Fat percentage (%) (r)	−0.324**	−0.317**	−0.366**	−0.233**
Muscle mass (kg) (r)	0.486**	0.443**	0.370**	0.426**
FFM (kg) (r)	0.429**	0.408**	0.280**	0.314**
TBW (%) (r)	0.610**	0.526**	0.491**	0.389**

Pearson Correlation Test, BMI: body mass index, FFM: fat-free mass, TBW: total body water, t0: preoperative, t1: postoperative 1. month, t2: 3. month, t3: 6. month, **p < 0.001. Bold *p*-values indicate statistical significance (*p* < 0.05).

A significant negative correlation was observed between the amount of change in PhA at 6 months and age (*r* = −0.283, *p* = 0.002). In contrast, there was a significant positive correlation between the change in PhA at 6 months and height (*r* = 0.261, *p* = 0.004). A borderline significant positive correlation was found between the amount of change in PhA at 6 months and the changes in weight (*r* = 0.180, *p* = 0.050) and BMI (*r* = 0.291, *p* = 0.001) at 6 months. No significant correlation was observed between the change in PhA at 6 months and the changes in body fat percentage or fat mass at 6 months (*p* > 0.05). However, a significant positive correlation was detected between the change in PhA at 6 months and the change in muscle mass (*r* = 0.212, *p* = 0.020) at 6 months. There was no significant correlation between the change in PhA at 6 months and the changes in FFM or TBW percentage at 6 months (*p* > 0.05). A significant positive correlation was observed between the change in PhA at 6 months and the changes in FPG (*r* = 0.255, *p* = 0.005) and HOMA-IR (*r* = 0.181, *p* = 0.048) at 6 months. No significant correlation was found between the change in PhA at 6 months and the changes in HbA1c, creatinine, urea, ALT, AST, GGT, HGB, HCT, total cholesterol, LDL, HDL, triglycerides, parathormone, or vitamin B_12_ at 6 months (*p* > 0.05). A significant negative correlation was found between the change in PhA at 6 months and changes in Ferritin (*r* = −0.358, *p* < 0.001), vitamin D (*r* = −0.234, *p* = 0.010), and excess weight loss (EWL) (*r* = −0.220, *p* = 0.016) at 6 months (Table 1).

Table 6 presents the results of the multivariate linear regression analysis conducted to identify factors independently associated with the 6-month change in PhA. The overall model was statistically significant (*F* = 6.961, p < 0.001) and explained 33.4% of the variance in PhA change (*R*^2^ = 0.334; adjusted *R*^2^ = 0.286). Among the variables included in the model, vitamin D level (*β* = −0.185; *p* = 0.020), and excess weight loss (EWL) (*β* = −0.472; *p* = 0.003) were identified as independently associated variables with 6-month PhA change. HOMA-IR (*β* = 0.199; *p* = 0.046) also reached statistical significance but with a *p*-value close to the 0.05 threshold. In contrast, age, sex, BMI, muscle mass, FPG, and ferritin level were not significantly associated with PhA change (*p* > 0.05). Multicollinearity diagnostics revealed that VIF values for all variables were below 5, indicating the absence of significant multicollinearity in the model.

**Table 6:** Multivariate linear regression analysis of variables associated with the 6-month change in PhA.

Variable	*R* ^2^	Adjusted *R*^2^	B	*β*	*p*	95% CI	VIF
Age (years)	0.334	0.286	0.000	−0.010	0.911	−0.008 to 0.007	1.281
Sex			−0.071	−0.080	0.467	−0.264 to 0.122	1.522
BMI (kg/m^2^)			−0.006	−0.053	0.709	−0.036 to 0.024	3.405
Muscle mass (kg)			−0.008	−0.119	0.183	−0.019 to 0.004	1.309
FPG (mg/dL)			−0.001	−0.043	0.684	−0.004 to 0.003	1.823
HOMA-IR			0.099	0.199	**0.046**	0.002 to 0.197	1.617
Ferritin (ng/mL)			0.000	−0.043	0.609	−0.001 to 0.001	1.184
Vitamin D (ng/mL)			−0.008	−0.185	**0.020**	−0.014 to −0.001	1.036
EWL (%)			−0.011	−0.472	**0.003**	−0.019 to −0.004	4.132
Multivariate linear regression	*F* = 6.961				*p* < 0.001		

B: unstandardized regression coefficient, β: standardized regression coefficient, CI: confidence interval, VIF: variance inflation factor, BMI: body mass index, HOMA-IR: homeostatic model assessment of insulin resistance, EWL: excess weight loss. Bold *p*-values indicate statistical significance (*p* < 0.05). Sex was coded as 1 = female, 2 = male.

## 4 Discussion

To the best of our knowledge, this study is one of the most comprehensive to evaluate changes in PhA during the early postoperative period, across a broad range of parameters, in patients undergoing MBS. Unlike previous studies, our study included TB patients (*n* = 114), and a small group of SG patients (*n* = 6). Consistent with findings in the literature, the main finding was a decrease in PhA in the postoperative period compared to the preoperative period in both groups. Given the marked imbalance in group sizes (TB *n* = 114 vs. SG *n* = 6), comparisons between the two surgical techniques should be interpreted as exploratory rather than confirmatory. Although no statistically significant differences were found between the surgical techniques, PhA values were consistently higher in the TB group across all time periods.

In this study, similar to other studies (18, 21, 22), we found a significant decrease in PhA that was not statistically significant between two surgical techniques. This result contrasts with previous research by Golzarand et al. (23) who reported improved postoperative nutritional status with two different techniques (RYGB and SG) and found that PhA significantly reduced in both groups. The discrepancy may be attributed to the uneven distribution of patients across surgical groups in our research, which limited our ability to detect significance in both groups.

Our findings align with previous studies reporting that PhA decreases with age (24–26). We observed that older age was associated with a larger decline in PhA (*r* = −0.283, *p* = 0.002; Table 1), consistent with the overall decline in PhA observed throughout the postoperative period (Table 3). These results are consistent with an age-related decline in cellular integrity and body cell mass, possibly reflecting age-related loss of muscle mass, though this study did not directly assess physical function, muscle strength, or other functional outcomes.

Interestingly, our analysis revealed a significant positive correlation between patient height and the amount of change in PhA at 6th months postoperatively. Although Gonzalez et al. (27) have hypothesized that height is related to FFM component - suggesting that similar amounts of FFM could result in variations in PhA according to the subject’s height - beside that, to our knowledge, no previous studies have specifically examined this correlation. This finding suggests that taller patients experienced a smaller decline in PhA following surgery (*r* = 0.261, *p* = 0.004), although the underlying mechanisms remain unclear.

In studies evaluating the associations between PhA and body composition, Teixeira et al. (28) assessed PhA in 169 out of 379 bariatric patient and observed a significant negative relationship between PhA and decreased weight, FM and fat percentage in men, while a significant positive relationship was found between PhA and muscle mass in women. Rapid weight loss after MBS leads to reduction in muscle mass (29). Since muscle tissue is a key reservoir of intracellular water, its loss may shift the fluid balance toward extracellular fluid and contribute to the decline in PhA (12). In a study conducted by Vassilev et al. (13) with 173 bariatric patients, they observed a significant reduction in FFM with decreased PhA as well as a correlation between PhA and weight loss in 6th and 12th month postoperatively. It indicates that the postoperative decrease in PhA is linked to weight loss which may be related to the reduction in fat percentage (28). In addition to the FFM and FM parameters typically assessed in body composition studies, a recent study has shown that muscle mass and muscle quality serve as more sensitive biomarkers for PhA (29). Our study aligns with the literature, demonstrating a positive correlation between the amount of change in PhA at 6th month postoperatively and body weight and BMI. According to the study by Westphal et al. (30), PhA tends to increase with BMI up to 35 kg/m^2^, but decreases when BMI exceeds 35 kg/m^2^; in both genders, PhA was negatively associated with BMI values greater than 40 kg/m^2^. In our study, all patients had a BMI greater than 35 kg/m^2^ preoperatively, and experienced weight loss and a consequent decrease in BMI after surgery. This may explain why a greater postoperative reduction in body weight and BMI was associated with a larger decline in PhA (*r* = 0.180, *p* = 0.050 and *r* = 0.291, *p* = 0.001, respectively). Furthermore, muscle mass was positively correlated (*p* < 0.05) with the PhA, supporting finding in the limited findings in the literature. In contrast, we found a significant negative correlation between EWL and the change in PhA, indicating that patients with greater excess weight loss experienced a larger decline in PhA at 6 months; this may be related to loss of muscle mass.

In cases of serious illness, the PhA value decreases due to weakened cell membrane integrity, a reduction in cell count, and alterations in hydration status (31). Studies have shown that PhA levels are lower in individuals with diabetes compared to healthy controls (32–34). Specifically, individuals with type 2 diabetes exhibit lower PhA at medium and high frequencies (which penetrate the cell membrane) and higher PhA at low frequencies (which do not); suggesting decreased cellular integrity and changes in fluid distribution (35). Age-related loss of muscle mass and strength can exacerbate insulin resistance, which may, in turn, explain an indirect relationship between PhA and insulin resistance (36). In the studies that evaluated individuals with type 2 diabetes such as Yücel et al. (33) reported a significant negative correlation between PhA and HbA1c. In contrast, Hu et al. (32) found a negative but non-significant relationship between PhA and HbA1c, and a positive relationship between PhA and FPG. Similarly, Curvello-Silva et al. (37), in a study of 141 severely obese patients, observed negative correlation between PhA and HbA1c, and a positive, though non-significant, correlation between PhA and HOMA-IR (*p* > 0.05). To our knowledge, there is limited research on the correlations of PhA with HbA1c, FPG and HOMA-IR in MBS patients. Therefore, we also investigated whether these parameters might affect PhA in early postoperative period. In our study, we did not find a significant correlation between the amount of change in PhA and HbA1c at 6 months. However, a greater postoperative reduction in FPG and HOMA-IR was associated with a larger decline in PhA (*r* = 0.255, *p* = 0.005 and *r* = 0.181, *p* = 0.048, respectively); the HOMA-IR association was of borderline significance and should be interpreted with caution. It should be noted that this pattern refers to the correlation between 6-month changes in these variables (Table 1), whereas PhA showed a negative correlation with FPG values at each individual time point (Table 7). This discrepancy may reflect the fact that HbA1c represents long-term glycemic control, whereas the parallel decline in FPG, HOMA-IR, and PhA during the early postoperative period may instead reflect shared underlying drivers—such as the magnitude of weight loss and metabolic adaptation—rather than a direct physiological relationship between glycemic improvement and PhA decline. This discrepancy also underscores the exploratory nature of these correlational findings, which should be confirmed in future prospective studies.

**Table 7:** The relationship between PhA and biochemical parameters in all periods.

	PhA			
	t0	t1	t2	t3
HbA1c (%) (r)	−0.138	−0.341**	−0.182*	−0.119
FPG (mg/dL) (r)	−0.214*	−0.426**	−0.230*	−0.126
HOMA-IR (%) (r)	−0.057	−0.196*	−0.054	0.139
Total cholesterol (mg/dL) (r)	0.018	−0.082	−0.022	0.003
HDL (mg/dL) (r)	−0.160	−0.111	−0.043	−0.092
LDL (mg/dL) (r)	0.145	0.018	0.019	0.062
Triglycerides (mg/dL) (r)	0.100	−0.136	−0.009	0.015
ALT (U/L) (r)	0.249**	0.187*	0.197*	0.124
AST (U/L) (r)	0.137	0.057	0.130	0.076
GGT (U/L) (r)	−0.077	−0.155	−0.089	−0.210*
Creatinine (mg/dL) (r)	0.050	0.068	0.099	−0.131
Urea (mg/dL) (r)	−0.178	−0.129	−0.004	−0.265**
Vitamin B12 (pg/mL) (r)	−0.013	−0.056	−0.103	−0.033
Vitamin D (ng/mL) (r)	0.217*	0.006	0.065	−0.179
Ferritin (ng/mL) (r)	0.318**	0.141	−0.040	−0.066
PTH (pg/mL) (r)	−0.355**	−0.226*	−0.300**	−0.203*
HGB (g/dL) (r)	0.297**	0.237**	0.253**	0.263**
HCT (%) (r)	0.211*	0.071	0.191*	0.018

Pearson Correlation Test, FPG: fasting plasma glucose, HCT: hematocrit, HDL: high-density lipoprotein, HGB: hemoglobin, HOMA-IR: homeostatic model assessment of insulin resistance, LDL: low-density lipoprotein, PTH: parathormone, t_0_: preoperative, t_1_: postoperative 1. month, t_2_: 3. month, t_3_: 6. month; r: correlation coefficient, *p < 0.05, **p < 0.01. Bold *p*-values indicate statistical significance (*p* < 0.05).

As a contribution to the literature, our study found a significant negative correlation between ferritin and vitamin D levels and PhA. Specifically, a smaller decrease in ferritin was associated with a larger decline in PhA (*r* = −0.358, *p* < 0.001), and a larger increase in vitamin D level was associated with a larger decline in PhA (*r* = −0.234, *p* = 0.010). To our knowledge, no studies in MBS patients have specifically examined the relationship between these nutrition-related biochemical parameters and PhA. However, in a study by Kim et al. (38) involving hemodialysis patients, a negative but non-significant correlation was observed between PhA and ferritin levels. Obesity is a metabolic disorder associated with chronic low-grade inflammation (39). In inflammatory conditions, ferritin levels may rise as an acute-phase reactant which can lead to cellular dysfunction and stress (40, 41). Ferritin may reflect underlying inflammatory processes rather than iron status alone and this may have affected PhA levels, potentially leading to decrease.

The metabolic abnormalities observed in obesity may be attributed to disruptions in adipose tissue metabolism (42). As an active endocrine organ, adipose tissue also regulates systemic metabolism through dynamic cellular responses (43). Vitamin D is a fat-soluble compound associated with energy metabolism. Adiposity affects the bioavailability of vitamin D, and therefore vitamin D deficiency may be associated with obesity (44). Additionally, body fat percentage decreases in response to vitamin D supplementation (45). Vitamin D also offers additional benefits in terms of its anti-inflammatory effects (46). Obesity-related inflammation may contribute to changes in vitamin D metabolism and a decrease in PhA values.

Phase angle (PhA) may serve as a potential adjunctive marker of nutritional status in patients undergoing MBS. Given its ability to reflect cellular integrity and body cell mass, a decline in PhA during the postoperative period may warrant closer clinical attention alongside conventional nutritional markers, although this study did not assess validated malnutrition criteria, dietary adherence, muscle strength, functional outcomes, quality of life, or clinical endpoints. Further prospective studies incorporating these outcomes are needed before PhA can be considered a confirmed early indicator of nutritional deterioration. Moreover, integrating PhA measurements into clinical practice may warrant further investigation as a complementary tool to guide timely and individualized dietetic interventions, enabling clinicians to optimize nutritional support and potentially improve patient outcomes.

This study has several limitations. First, the retrospective design limits the ability to establish causal relationships. Second, the uneven distribution of patients between surgical groups, particularly the small number of SG patients, reduces the statistical power for direct comparisons. Third, only patients who regularly attended follow-up visits and had complete data available were included, which may introduce selection bias. The characteristics of excluded patients could not be examined; therefore, the included patients may differ systematically from those excluded in terms of sociodemographic and clinical characteristics, and the generalizability of the findings to the broader MBS population may be limited. Accordingly, these findings should be interpreted as applicable to patients who completed structured follow-up, not the broader MBS population, given the potential for selection bias.

Additionally, due to the retrospective nature of the study design, potential confounding factors such as medications, individual dietary intake, actual caloric and protein intake, physical activity, and hydration status could not be objectively quantified, which may have influenced the observed variability in body composition and PhA outcomes. The use of a standardized postoperative nutritional protocol may also have masked individual variability in nutritional responses. Furthermore, the large number of correlations may increase the risk of Type I error; additionally, some correlations were weak. Therefore, statistically significant findings should be interpreted with caution and confirmed in future prospective studies. Future prospective studies with balanced groups and individualized nutritional monitoring are warranted to better elucidate these relationships.

## 5 Conclusion

In conclusion, this study suggests that PhA significantly decreases during the early postoperative period following MBS, and this change appears to be associated with body composition including body weight, BMI, and muscle mass, as well as metabolic parameters such as FPG, ferritin, and vitamin D. The findings suggest that PhA may serve as a potential adjunctive marker reflecting early nutritional and metabolic adaptations, although further prospective studies incorporating validated clinical and functional outcomes are needed to confirm its utility. These associations suggest that incorporating PhA into routine follow-up may warrant further investigation as a complementary tool, potentially contributing to the assessment of patient outcomes and personalized nutritional management strategies, pending confirmation in future prospective studies. Future studies with larger, well-balanced cohorts and individualized nutritional interventions are needed to further clarify the determinants and clinical significance of PhA changes in this MBS population.

## Data Availability

The original contributions presented in the study are included in the article/Supplementary material. Further inquiries can be directed to the corresponding author, subject to institutional and ethics committee restrictions.
