# Supplementary material for: Phase angle as a potential marker of early nutritional and metabolic changes following metabolic bariatric surgery: a retrospective study
Source: Front Nutr. 2026 Sep 9;13:1853597. doi: 10.3389/fnut.2026.1853597 (PMC13597673; doi:10.3389/fnut.2026.1853597)
Supplement: Supplementary file 1 [file Table_1.DOCX]

**Supplementary Table**

Table 1. The variation of biochemical parameters and anthropometric measurements

|  |  | TB (n:114) | | | |  | SG (n:6) | | | | p | |
| --- | --- | --- | --- | --- | --- | --- | --- | --- | --- | --- | --- | --- |
|  |  | Mean.±SD | | | Median |  | Mean.±SD | | | Median |  |  |
| ***Weight (kg)*** | |  |  |  |  |  |  |  |  |  |  |  |
| t0 | | 110.8 | ± | 17.9 | 110.1 |  | 133.2 | ± | 35.2 | 139.8 | 0.095 | ^m^ |
| t1 | | 99.3 | ± | 16.1 | 97.7 |  | 118.6 | ± | 31.4 | 120.5 | 0.099 | ^m^ |
| t2 | | 91.0 | ± | 14.5 | 89.8 |  | 110.6 | ± | 27.5 | 114.5 | 0.081 | ^m^ |
| t3 | | 84.4 | ± | 13.6 | 84.8 |  | 101.8 | ± | 27.2 | 105.5 | 0.090 | ^m^ |
| ***BMI (kg/m^2^)*** | |  |  |  |  |  |  |  |  |  |  |  |
| t0 | | 40.4 | ± | 4.7 | 38.9 |  | 46.6 | ± | 6.8 | 46.7 | ***0.016*** | ^m^ |
| t1 | | 36.2 | ± | 4.4 | 34.5 |  | 41.5 | ± | 5.7 | 41.0 | ***0.014*** | ^m^ |
| t2 | | 33.2 | ± | 4.0 | 32.5 |  | 38.8 | ± | 6.0 | 38.8 | ***0.016*** | ^m^ |
| t3 | | 30.8 | ± | 3.9 | 30.4 |  | 35.7 | ± | 6.4 | 35.9 | ***0.037*** | ^m^ |
| ***Fat percentage (%)*** | |  |  |  |  |  |  |  |  |  |  |  |
| t0 | | 44.8 | ± | 6.1 | 45.5 |  | 49.8 | ± | 5.1 | 50.4 | 0.062 | ^m^ |
| t1 | | 42.5 | ± | 7.4 | 43.9 |  | 50.3 | ± | 5.9 | 49.9 | ***0.018*** | ^m^ |
| t2 | | 38.0 | ± | 8.4 | 39.0 |  | 45.9 | ± | 5.8 | 44.8 | ***0.022*** | ^m^ |
| t3 | | 33.6 | ± | 9.2 | 33.8 |  | 42.6 | ± | 7.2 | 41.1 | ***0.026*** | ^m^ |
| ***Fat mass (kg)*** | |  |  |  |  |  |  |  |  |  |  |  |
| t0 | | 49.4 | ± | 10.5 | 46.5 |  | 65.9 | ± | 17.2 | 64.2 | ***0.010*** | ^m^ |
| t1 | | 42.2 | ± | 10.5 | 39.9 |  | 59.7 | ± | 17.4 | 57.3 | ***0.006*** | ^m^ |
| t2 | | 34.6 | ± | 9.6 | 32.9 |  | 50.7 | ± | 13.7 | 50.7 | ***0.005*** | ^m^ |
| t3 | | 28.3 | ± | 9.2 | 27.4 |  | 43.4 | ± | 13.8 | 41.8 | ***0.009*** | ^m^ |
| ***Muscle Mass (kg)*** | |  |  |  |  |  |  |  |  |  |  |  |
| t0 | | 29.7 | ± | 7.1 | 28.8 |  | 32.3 | ± | 13.1 | 31.4 | 0.866 | ^m^ |
| t1 | | 26.7 | ± | 6.6 | 26.7 |  | 28.3 | ± | 10.5 | 27.1 | 0.805 | ^m^ |
| t2 | | 25.8 | ± | 6.7 | 25.5 |  | 28.2 | ± | 10.0 | 26.8 | 0.580 | ^m^ |
| t3 | | 24.9 | ± | 6.8 | 23.7 |  | 26.7 | ± | 10.1 | 25.4 | 0.691 | ^m^ |
| ***FFM (kg)*** | |  |  |  |  |  |  |  |  |  |  |  |
| t0 | | 61.2 | ± | 12.4 | 60.1 |  | 67.3 | ± | 20.6 | 68.9 | 0.523 | ^m^ |
| t1 | | 57.1 | ± | 11.7 | 55.5 |  | 58.9 | ± | 17.0 | 58.5 | 0.885 | ^m^ |
| t2 | | 56.2 | ± | 12.4 | 54.0 |  | 59.9 | ± | 17.0 | 58.9 | 0.613 | ^m^ |
| t3 | | 55.6 | ± | 13.0 | 52.9 |  | 58.4 | ± | 17.5 | 58.5 | 0.736 | ^m^ |
| ***TBW (%)*** | |  |  |  |  |  |  |  |  |  |  |  |
| t0 | | 40.9 | ± | 4.6 | 40.4 |  | 35.2 | ± | 6.2 | 36.6 | ***0.037*** | ^m^ |
| t1 | | 42.2 | ± | 5.2 | 41.6 |  | 36.7 | ± | 3.9 | 37.1 | ***0.012*** | ^m^ |
| t2 | | 45.3 | ± | 6.0 | 44.6 |  | 39.9 | ± | 4.0 | 41.0 | ***0.026*** | ^m^ |
| t3 | | 48.2 | ± | 6.5 | 48.7 |  | 41.9 | ± | 4.9 | 43.4 | ***0.029*** | ^m^ |
| ***HbA1c (%)*** | |  |  |  |  |  |  |  |  |  |  |  |
| t0 | | 7.6 | ± | 2.0 | 7.1 |  | 8.4 | ± | 3.7 | 7.2 | 0.928 | ^m^ |
| t1 | | 6.5 | ± | 1.3 | 6.1 |  | 7.4 | ± | 2.0 | 6.9 | 0.309 | ^m^ |
| t2 | | 5.9 | ± | 0.9 | 5.8 |  | 6.2 | ± | 1.2 | 5.9 | 0.643 | ^m^ |
| t3 | | 5.8 | ± | 0.9 | 5.7 |  | 6.2 | ± | 0.8 | 5.9 | 0.206 | ^m^ |
| ***FPG (mg/dL)*** | |  |  |  |  |  |  |  |  |  |  |  |
| t0 | | 158.7 | ± | 71.8 | 138.6 |  | 169.8 | ± | 101.3 | 141.7 | 0.866 | ^m^ |
| t1 | | 109.1 | ± | 39.8 | 98.7 |  | 117.8 | ± | 45.9 | 98.4 | 0.786 | ^m^ |
| t2 | | 107.1 | ± | 39.6 | 93.5 |  | 105.6 | ± | 27.0 | 95.6 | 0.847 | ^m^ |
| t3 | | 100.7 | ± | 28.9 | 91.1 |  | 113.6 | ± | 35.7 | 107.9 | 0.434 | ^m^ |
| ***HOMA-IR*** | |  |  |  |  |  |  |  |  |  |  |  |
| t0 | | 9.0 | ± | 9.0 | 6.6 |  | 6.1 | ± | 3.1 | 7.1 | 0.434 | ^m^ |
| t1 | | 2.7 | ± | 2.1 | 2.1 |  | 3.0 | ± | 2.3 | 2.0 | 0.871 | ^m^ |
| t2 | | 2.3 | ± | 1.4 | 2.0 |  | 4.9 | ± | 7.0 | 2.2 | 0.634 | ^m^ |
| t3 | | 1.8 | ± | 0.9 | 1.6 |  | 2.2 | ± | 0.8 | 2.3 | 0.189 | ^m^ |
| ***Creatinine (mg/dL)*** | |  |  |  |  |  |  |  |  |  |  |  |
| t0 | | 0.79 | ± | 0.21 | 0.78 |  | 0.98 | ± | 0.43 | 0.84 | 0.312 | ^m^ |
| t1 | | 0.79 | ± | 0.22 | 0.78 |  | 0.80 | ± | 0.36 | 0.77 | 0.575 | ^m^ |
| t2 | | 0.72 | ± | 0.18 | 0.70 |  | 0.74 | ± | 0.30 | 0.64 | 0.669 | ^m^ |
| t3 | | 0.71 | ± | 0.20 | 0.66 |  | 0.82 | ± | 0.51 | 0.63 | 0.634 | ^m^ |
| ***Urea (mg/dL)*** | |  |  |  |  |  |  |  |  |  |  |  |
| t0 | | 29.6 | ± | 8.9 | 28.0 |  | 39.4 | ± | 22.5 | 32.4 | 0.309 | ^m^ |
| t1 | | 23.6 | ± | 10.4 | 21.9 |  | 26.2 | ± | 12.8 | 21.6 | 0.704 | ^m^ |
| t2 | | 24.4 | ± | 8.5 | 23.2 |  | 23.4 | ± | 7.1 | 22.9 | 0.847 | ^m^ |
| t3 | | 25.2 | ± | 9.1 | 23.5 |  | 32.8 | ± | 29.6 | 25.5 | 0.481 | ^m^ |
| ***ALT (U/L)*** | |  |  |  |  |  |  |  |  |  |  |  |
| t0 | | 40.8 | ± | 34.0 | 31.0 |  | 26.8 | ± | 13.5 | 23.0 | 0.159 | ^m^ |
| t1 | | 34.0 | ± | 21.7 | 28.5 |  | 27.6 | ± | 9.9 | 24.7 | 0.555 | ^m^ |
| t2 | | 25.2 | ± | 15.1 | 20.0 |  | 18.7 | ± | 6.2 | 18.0 | 0.257 | ^m^ |
| t3 | | 22.7 | ± | 11.7 | 20.0 |  | 14.2 | ± | 4.9 | 13.5 | ***0.026*** | ^m^ |
| ***AST (U/L)*** | |  |  |  |  |  |  |  |  |  |  |  |
| t0 | | 29.0 | ± | 18.7 | 24.6 |  | 23.5 | ± | 15.0 | 17.4 | 0.116 | ^m^ |
| t1 | | 26.8 | ± | 11.7 | 23.2 |  | 25.7 | ± | 7.9 | 21.9 | 0.986 | ^m^ |
| t2 | | 23.1 | ± | 11.8 | 20.0 |  | 18.6 | ± | 2.0 | 18.7 | 0.341 | ^m^ |
| t3 | | 20.9 | ± | 7.9 | 19.1 |  | 15.8 | ± | 3.8 | 14.3 | ***0.041*** | ^m^ |
| ***GGT (U/L)*** | |  |  |  |  |  |  |  |  |  |  |  |
| t0 | | 52.0 | ± | 58.5 | 37.0 |  | 57.5 | ± | 62.5 | 30.6 | 0.876 | ^m^ |
| t1 | | 31.3 | ± | 34.3 | 21.9 |  | 45.0 | ± | 63.8 | 19.4 | 0.866 | ^m^ |
| t2 | | 26.7 | ± | 35.6 | 17.6 |  | 72.0 | ± | 129.6 | 22.1 | 0.539 | ^m^ |
| t3 | | 24.4 | ± | 23.0 | 16.7 |  | 39.1 | ± | 63.1 | 14.2 | 0.754 | ^m^ |
| ***HGB (g/dL)*** | |  |  |  |  |  |  |  |  |  |  |  |
| t0 | | 14.0 | ± | 1.8 | 14.0 |  | 13.3 | ± | 2.1 | 13.9 | 0.500 | ^m^ |
| t1 | | 13.8 | ± | 1.4 | 13.8 |  | 13.8 | ± | 1.7 | 14.5 | 0.772 | ^m^ |
| t2 | | 14.3 | ± | 3.3 | 14.0 |  | 13.6 | ± | 1.5 | 13.5 | 0.473 | ^m^ |
| t3 | | 14.3 | ± | 3.2 | 14.0 |  | 14.1 | ± | 1.0 | 14.4 | 0.899 | ^m^ |
| ***HCT (%)*** | |  |  |  |  |  |  |  |  |  |  |  |
| t0 | | 40.9 | ± | 6.0 | 41.6 |  | 39.4 | ± | 4.6 | 40.7 | 0.396 | ^m^ |
| t1 | | 40.4 | ± | 4.2 | 40.7 |  | 40.9 | ± | 3.6 | 42.5 | 0.700 | ^m^ |
| t2 | | 40.9 | ± | 3.8 | 40.8 |  | 40.7 | ± | 3.6 | 41.2 | 0.942 | ^m^ |
| t3 | | 43.7 | ± | 31.6 | 40.5 |  | 41.3 | ± | 1.7 | 42.1 | 0.551 | ^m^ |
| ***Total Cholesterol (mg/dL)*** | |  |  |  |  |  |  |  |  |  |  |  |
| t0 | | 210.5 | ± | 54.7 | 206.2 |  | 206.7 | ± | 33.2 | 196.4 | 0.942 | ^m^ |
| t1 | | 173.0 | ± | 38.1 | 170.4 |  | 202.0 | ± | 53.3 | 202.9 | 0.155 | ^m^ |
| t2 | | 173.3 | ± | 42.0 | 170.5 |  | 210.5 | ± | 67.0 | 205.0 | 0.120 | ^m^ |
| t3 | | 159.9 | ± | 42.7 | 152.3 |  | 198.4 | ± | 37.8 | 188.9 | ***0.018*** | ^m^ |
| ***LDL (mg/dL)*** | |  |  |  |  |  |  |  |  |  |  |  |
| t0 | | 135.3 | ± | 43.1 | 129.2 |  | 131.0 | ± | 31.8 | 129.2 | 0.928 | ^m^ |
| t1 | | 114.9 | ± | 37.9 | 111.1 |  | 149.1 | ± | 54.6 | 153.3 | 0.088 | ^m^ |
| t2 | | 116.2 | ± | 98.7 | 101.6 |  | 132.0 | ± | 52.5 | 129.5 | 0.198 | ^m^ |
| t3 | | 98.6 | ± | 34.7 | 91.8 |  | 122.4 | ± | 19.1 | 118.0 | ***0.028*** | ^m^ |
| ***HDL (mg/dL)*** | |  |  |  |  |  |  |  |  |  |  |  |
| t0 | | 48.1 | ± | 12.2 | 45.1 |  | 50.7 | ± | 9.8 | 52.3 | 0.516 | ^m^ |
| t1 | | 42.1 | ± | 9.3 | 40.2 |  | 43.5 | ± | 8.6 | 44.1 | 0.639 | ^m^ |
| t2 | | 41.1 | ± | 8.9 | 40.0 |  | 42.2 | ± | 13.4 | 39.3 | 0.899 | ^m^ |
| t3 | | 40.7 | ± | 10.5 | 39.3 |  | 40.3 | ± | 6.9 | 37.9 | 0.942 | ^m^ |
| ***Triglycerides (mg/dL)*** | |  |  |  |  |  |  |  |  |  |  |  |
| t0 | | 213.8 | ± | 132.8 | 176.5 |  | 152.8 | ± | 109.0 | 98.4 | 0.119 | ^m^ |
| t1 | | 137.8 | ± | 52.3 | 126.4 |  | 134.7 | ± | 50.6 | 118.8 | 0.880 | ^m^ |
| t2 | | 134.9 | ± | 62.0 | 120.8 |  | 145.4 | ± | 32.6 | 141.1 | 0.300 | ^m^ |
| t3 | | 122.9 | ± | 52.3 | 112.8 |  | 145.1 | ± | 71.5 | 123.1 | 0.539 | ^m^ |
| ***Ferritin (ng/mL)*** | |  |  |  |  |  |  |  |  |  |  |  |
| t0 | | 89.1 | ± | 96.7 | 48.7 |  | 66.6 | ± | 45.9 | 68.5 | 0.923 | ^m^ |
| t1 | | 95.3 | ± | 93.8 | 66.4 |  | 130.5 | ± | 116.8 | 124.6 | 0.647 | ^m^ |
| t2 | | 65.5 | ± | 70.1 | 42.0 |  | 96.0 | ± | 89.8 | 88.5 | 0.492 | ^m^ |
| t3 | | 61.6 | ± | 62.4 | 46.0 |  | 84.3 | ± | 79.8 | 78.5 | 0.763 | ^m^ |
| ***PTH (pg/mL)*** | |  |  |  |  |  |  |  |  |  |  |  |
| t0 | | 61.7 | ± | 30.6 | 54.9 |  | 98.7 | ± | 62.6 | 76.6 | 0.082 | ^m^ |
| t1 | | 57.6 | ± | 27.7 | 50.8 |  | 74.2 | ± | 41.2 | 67.2 | 0.258 | ^m^ |
| t2 | | 56.2 | ± | 22.9 | 51.8 |  | 77.2 | ± | 55.8 | 48.8 | 0.567 | ^m^ |
| t3 | | 57.3 | ± | 28.7 | 53.9 |  | 57.6 | ± | 24.7 | 45.6 | 0.933 | ^m^ |
| ***Vitamin B12*** | |  |  |  |  |  |  |  |  |  |  |  |
| t0 | | 477.2 | ± | 320.6 | 385.5 |  | 427.0 | ± | 144.7 | 372.5 | 0.966 | ^m^ |
| t1 | | 469.7 | ± | 200.9 | 414.0 |  | 592.7 | ± | 229.4 | 563.0 | 0.097 | ^m^ |
| t2 | | 414.9 | ± | 158.6 | 382.5 |  | 494.2 | ± | 94.4 | 476.5 | 0.075 | ^m^ |
| t3 | | 463.2 | ± | 203.5 | 420.5 |  | 368.7 | ± | 61.9 | 360.5 | 0.379 | ^m^ |
| ***Vitamin D*** | |  |  |  |  |  |  |  |  |  |  |  |
| t0 | | 14.7 | ± | 8.1 | 13.1 |  | 7.9 | ± | 3.2 | 8.0 | ***0.009*** | ^m^ |
| t1 | | 32.0 | ± | 30.4 | 29.0 |  | 19.7 | ± | 5.1 | 18.4 | ***0.007*** | ^m^ |
| t2 | | 28.9 | ± | 13.3 | 28.0 |  | 25.8 | ± | 7.0 | 25.6 | 0.406 | ^m^ |
| t3 | | 26.9 | ± | 11.0 | 26.7 |  | 29.6 | ± | 8.3 | 31.7 | 0.300 | ^m^ |
| ***EWL (%)*** | |  |  |  |  |  |  |  |  |  |  |  |
| t1 | | 28.4 | ± | 9.8 | 28.5 |  | 24.1 | ± | 6.3 | 22.0 | 0.215 | ^m^ |
| t2 | | 48.3 | ± | 13.4 | 47.2 |  | 37.1 | ± | 15.2 | 32.8 | 0.077 | ^m^ |
| t3 | | 64.3 | ± | 18.0 | 64.1 |  | 52.4 | ± | 23.0 | 44.1 | 0.099 | ^m^ |
| ^m^ Mann-Whitney U Test / ^w^ Wilcoxon Test; BMI: body mass index, EWL: extra weight loss, FFM: fat-free mass, FPG: fasting plasma glucose, HDL: high-density lipoprotein, LDL: low-density lipoprotein, PTH: parathormone, HGB: hemoglobin, HCT: hematocrit t_0_: preoperative, TBW: total body water, t_1_: postoperative 1. month, t_2_: 3. month, t_3_: 6. month | | | | | | | | | | | | |
